# Analytic descriptions of cylindrical electromagnetic waves in a nonlinear medium

**DOI:** 10.1038/srep11071

**Published:** 2015-06-15

**Authors:** Hao Xiong, Liu-Gang Si, Xiaoxue Yang, Ying Wu

**Affiliations:** 1 Wuhan National Laboratory for Optoelectronics and School of Physics, Huazhong University of Science and Technology, Wuhan 430074, China

## Abstract

A simple but highly efficient approach for dealing with the problem of cylindrical electromagnetic waves propagation in a nonlinear medium is proposed based on an exact solution proposed recently. We derive an analytical explicit formula, which exhibiting rich interesting nonlinear effects, to describe the propagation of any amount of cylindrical electromagnetic waves in a nonlinear medium. The results obtained by using the present method are accurately concordant with the results of using traditional coupled-wave equations. As an example of application, we discuss how a third wave affects the sum- and difference-frequency generation of two waves propagation in the nonlinear medium.

Superposition principle is the fundamental feature of linear optics. Using the superposition principle, complex light fields can be decomposed into simple light fields, and a lot of effective research methods, such as spectrum analysis method and Green’s function method, come up. However, linear media give only an approximation of the real media. The real media are usually nonlinear. This makes wave propagation in nonlinear media to be a fundamental problem and central issue in physics, and a lot of interesting phenomena occur when the dielectric susceptibility of a medium is not a linear function of the electric field amplitude^1^. One of the typical phenomena, second-harmonic generation, was first observed in quartz^2^, and a phenomenological approach to describe nonlinear optics phenomena by using coupled-wave equations was developed in the 1960s^3^,^4^. In the following 50 years, nonlinear optics become one of the most active research areas and a number of important features of nonlinear optics have been found, including nonlinear optics with few-cycle^5^ or Single-cycle^6^ laser fields, nonlinear optics at low light levels^7^, surface nonlinear optics^8^, nonlinear optics in photonic nanowires^9^, cylindrical nonlinear optics^10^, nonlinear optics with semiconductor microcavities^11^, nonlinear optics in the extreme ultraviolet^12^, optomechanical nonlinear optics^13^, solitons^14^, and nonlinear optics in nanostructures^15^. In a general way, analytical methods based on the superposition principle are difficult to build in nonlinear optics. Numerical methods become an important tool for dealing with problems of electromagnetic waves propagation in a nonlinear medium^5^.

Electromagnetic waves with cylindrical symmetry are always studied in linear media, such as electromagnetic scattering^16^,^17^ and optical cloaking^18^. The features of cylindrical electromagnetic waves in a nonlinear medium, however, remain poorly studied^19^. In this work, we present a simple but highly efficient approach to deal with interactions between any amount of cylindrical electromagnetic waves in a nonlinear medium. Obtained explicit analytical expressions reproduce accurately the results of using the coupled-wave equations. Our work is an interesting extension of the recent publication^19^, which put forward an important technique to construct exact axisymmetric solutions of Maxwells equations in a nonlinear nondispersive medium. As a new exact solution, it is useful and interesting to find and examine the physical nature contains in it. We show that this solution can be used to deal with the problems of cylindrical electromagnetic waves propagation in a nonlinear medium, and nonlinear optical effects, such as wave mixing, come out quite naturally from the exact solution. As an example of application, we show that sum- and difference-frequency generation of two waves propagation in a nonlinear medium is affected by a third wave.

## Results

### Axisymmetric cylindrical electromagnetic model

We shall introduce the physical model discussed in this work. Considering the medium possesses an axis of symmetry which is taken as the *z* axis of a cylindrical coordinate system (*r*, *ϕ*, *z*), we use the axisymmetric model in which the fields are independent of *ϕ* and *z*, then the Maxwell equations can be written as follows^19^:


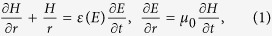


where *H* ≡ *H*_*ϕ*_(*r*, *t*), *E* ≡ *E*_*z*_(*r*, *t*), *ε*(*E*) = d*D*/d*E* = *ε*_*0*_*ε*_*1*_exp(*αE*), with *ε*_1_ and *α* are certain constants. Such model describes cylindrical electromagnetic waves propagation in a nonlinear medium, and some works^19^,^20^,^21^,^22^,^23^ have been done in this topic. Exact solution of such system has been obtained by using an interesting technique^19^:


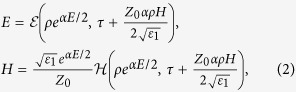


where *ρ* = *r*/*a*, 
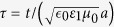
, 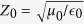 and *a* is a constant with the dimension of length. 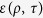 and 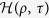 represent the solution of linear problem, for more clearly Eqs. (1) with *α* = 0, in variables 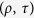.

### Exact solutions and explicit analytical expressions

Now we will use this exact solution to deal with problems of interactions between cylindrical electromagnetic waves in a nonlinear medium. The solution of single cylindrical wave propagation in an infinite and linear medium is: 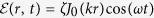 and 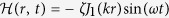. Here *J*_*m*_ is a Bessel function of the first kind of order *m*, *ζ* is an amplitude constant, and 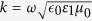 is the wave number. For linear medium, the superposition principle is always applicable. Now we will use the superposition principle to obtain the exact solutions of the nonlinear case accordingly. Starting from the superposition principle, the exact solution of the linear problem can be easily extended to describe cylindrical waves in the medium:


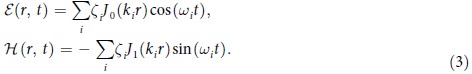


Rewriting it in variables 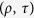, the solution becomes: 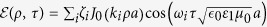 and 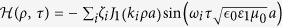. By using Eqs. (2) we can obtain the solution of the nonlinear problem:


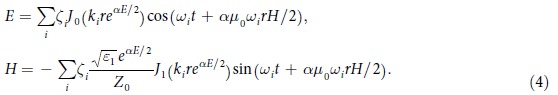


This solution is in implicit form, describes cylindrical electromagnetic waves in a nonlinear medium, and shows that the electric field and magnetic field of all the cylindrical electromagnetic waves in the nonlinear medium are not separate, but coupling with each other by nonlinear coefficient *α*. If *α* → 0, obviously Eqs. (4) will go to the linear case (3) and the coupling between the electric field and magnetic field of all the cylindrical electromagnetic waves will be disappeared.

Interactions between cylindrical electromagnetic waves in a nonlinear medium have been shown in Eqs. (4), however, it is difficult to use this exact solution to analyse nonlinear interactions and phenomena. In what follows, we will propose a method to obtain an analytical explicit formula from the exact solution (4), and from the analytical explicit formula we can know some more details of such physical process.

We consider that there are *q* fundamental electromagnetic waves in the nonlinear medium and |*αE*/2| << 1 where *q* is an arbitrary number. After some deductions (see Section Methods), we obtain:


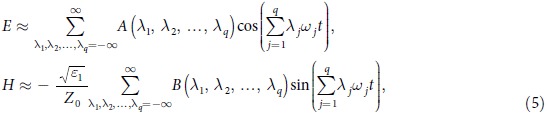


where
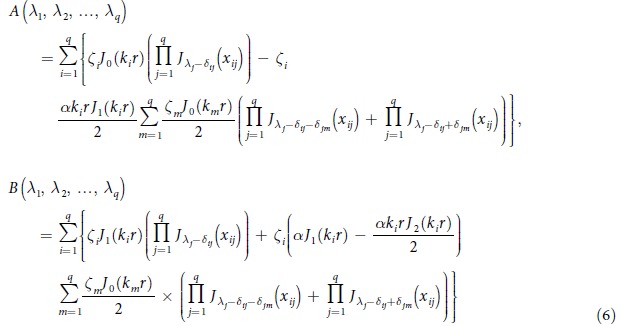

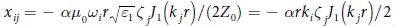
 and *δ*_*ij*_ is the Kronecker Delta. Physical significance of Eqs. (5) is that if *q* electromagnetic waves with different frequencies propagate in a nonlinear medium, then there are frequency mixing generation and the amplitude of electric field with frequency 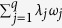 is *A*(*λ*_1_,*λ*_2_,…,*λ*_*q*_).

For the case *α* = 0, then *x*_*ij*_ = 0, and Eqs. (5) become


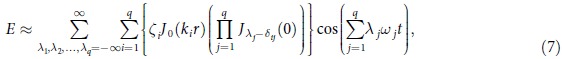



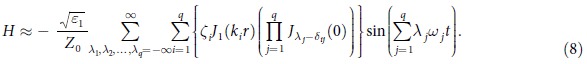


Using the feature of Bessel functions


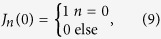
we can see that 
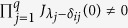
 requires that *λ*_*j*_−*δ*_*ij*_ = 0 for 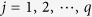. Then Eqs. (7) and (8) become


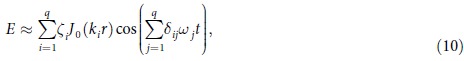



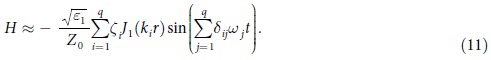


Equations (10) and (11) are exactly the linear solution (19) and (20), which verifies that Eqs. (5) go into the linear case (19) and (20) when *α* = 0.

By using Eqs. (5), one can obtain the amplitude of the electric and magnetic field of various nonlinear effects, such as second harmonic, sum frequency and difference frequency generation. In what follows, we will give some examples.

## Discussion

### Comparison of the calculations with traditional coupled-wave equations

Reference^21^ have used this method to study second-harmonic generation and shown that second-harmonic generation comes out quite naturally from the exact solutions (2). There are higher harmonics due to existence of sum frequency and difference frequency of base frequency and second-harmonic. Here we will give a detailed discussion by using Eqs. (5). In the case of *q* = 1, Eqs. (5) become:


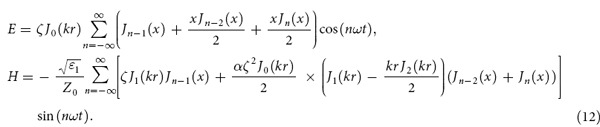
where *x* = −*αrkζJ*_1_(*kr*)/2. It can be found that there are second and higher harmonics exist in the medium. To describe the amplitude of cylindrical second-harmonic generation, the component of negative frequency in Eqs. (12) should also be considered.

From a different point of view, such second-harmonic generation also can be described by the cylindrical coupled-wave equations^21^,^22^:


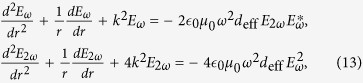
where *E*_*ω*_ and *E*_2*ω*_ present the electric field amplitude of the wave with frequency *ω* and 2*ω*, respectively. *d*_eff_ is effective second order nonlinear optical coefficient of the nonlinear medium, and the relationship between *α* and *d*_eff_ is *α* = 2*d*_eff_, which have been obtained previously^21^. The initial condition of equations (13) is


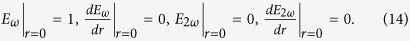


It means that at *r* = 0, there is only cylindrical electromagnetic wave with fundamental frequency *ω* and unit amplitude, and equations (13) describe the amplitude of cylindrical second-harmonic generation at arbitrary *r*, which can be solved by Runge-Kutta method.

**Figure 1:**
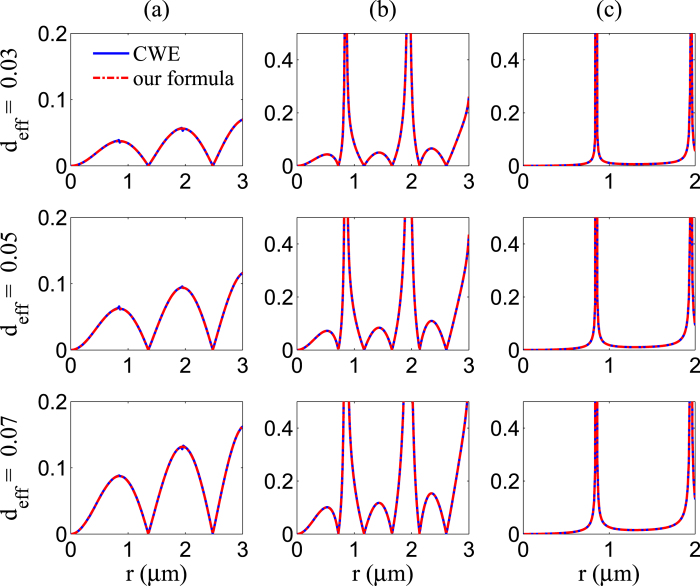
Comparison diagram of using coupled-wave equations (CWE) and the analytical explicit formula. The blue solid curve presents calculation results of using coupled-wave equations while the red dashed curve presents calculation results of using the analytical explicit formula. Efficiencies of generation of (**a**) second harmonic, (**b**) sum frequency and (**c**) difference frequency with different nonlinear coefficient have been shown. We use *ζ*_1_ = *ζ*_2_ = 1 and *ε*_1_ = 2. In Fig. (**a**) the fundamental frequency is *ω* = 6 × 10^8^ MHz, and in Fig. (**b**) and (**c**) the fundamental frequencies are *ω*_1_ = 6 × 10^8^ MHz and *ω*_2_ = 8 × 10^8^ MHz.

Figure 1(a) shows a comparison of using the coupled-wave equations and the analytical explicit formula (5) or (12) to calculate efficiencies of second-harmonic generation which is defined as *η* = *E*_2*ω*_/*E*_*ω*_, and we can find that the results obtained by the two methods are concordant with each other.

Further more, if we consider that there are two fundamental electromagnetic waves with frequencies *ω*_1_ and *ω*_2_ propagating in the nonlinear medium, then *q* = 2 and Eqs. (5) can be expressed as:


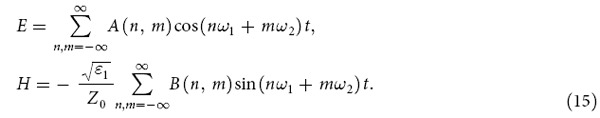


Equations (15) contains second and higher harmonic generation, sum- and difference-frequency, and even frequency mixing. We use *E*_*ω*1_, *E*_*ω*2_, *E*_*ω*1+*ω*2_, and *E*_*ω*1−*ω*2_ to present the electric field amplitude of the wave with frequency *ω*_1_, *ω*_2_, *ω*_1_ + *ω*_2_ and *ω*_1_−*ω*_2_, respectively. *E*_*ω*1_, *E*_*ω*2_, *E*_*ω*1+*ω*2_, and *E*_*ω*1−*ω*2_ can be easily obtained from Eqs. (15): *E*_*ω*1_ = *A*(1,0) + *A*(−1,0), *E*_*ω*2_ = *A*(0,1) + *A*(0,−1), *E*_*ω*1+*ω*2_=*A*(1,1) + *A*(−1,−1), and 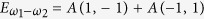, where *A*(*n,m*) have been given in equations (6).

Sum- and difference-frequency generation can also be described by the coupled-wave equations^22^. The coupled-wave equations which describe the sum-frequency generation are


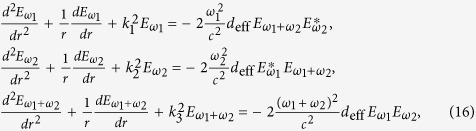
while describe the difference-frequency generation are


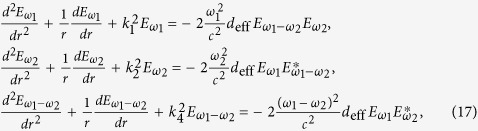
where *k*_3 _=_ _*k*_1_ + *k*_2_ and *k*_4_ = *k*_1_ − *k*_2_. Figure 1(b,c) shows a comparison of using coupled-wave equations and the analytical explicit formula (5) or (15) to calculate efficiencies of sum and difference frequency generation, which are defined as 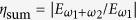 and 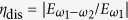. We can find that, the same as the second harmonic generation case, the results obtained by two methods are concordant with each other.

### Interactions between cylindrical electromagnetic waves

**Figure 2:**
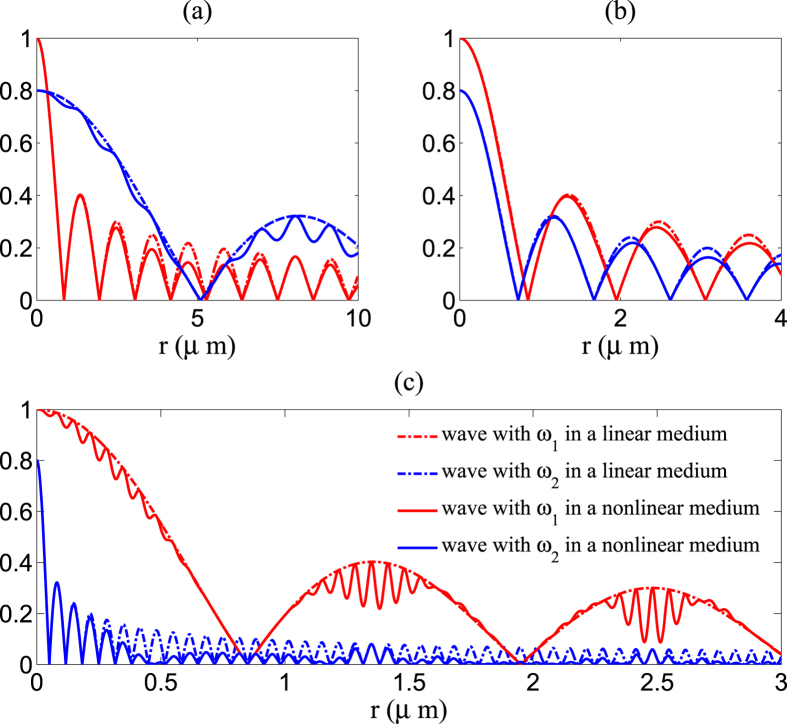
Comparison diagram of cylindrical electromagnetic waves propagation in a linear and nonlinear medium. We consider that there are two waves propagate in the medium and we use *ζ*_1_ = 1, *ζ*_2_ = 0.8, *α* = 0.4, and *ε*_1_ = 2 in equations (6). The red solid curve presents calculation results of 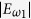 in the nonlinear medium, the blue solid curve presents calculation results of 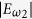 in the nonlinear medium, the red dashed curve presents calculation results of 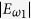 in the linear medium and the blue dashed curve presents calculation results of 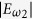 in the linear medium. We fix *ω*_1_ = 6 × 10^8^ MHz, and use (**a**) *ω*_2_ = 1 × 10^8^ MHz, (**b**) *ω*_2_ = 7 × 10^8^ MHz and (**c**) *ω*_2_ = 100 × 10^8^ MHz.

Now we will use the analytical explicit formula (5) to discuss some more interactions between cylindrical electromagnetic waves in a nonlinear medium. Figure 2 give a comparison of cylindrical electromagnetic waves propagation in a linear medium and in a nonlinear medium. We fix *ω*_1_ = 6 × 10^8^ MHz, and use three different *ω*_2_. We consider that different *ω*_2_ will lead to different effects via interactions between cylindrical electromagnetic waves. Figure 2(a) shows the results of interactions between wave with *ω*_1_ = 6 × 10^8^ MHz and wave with lower frequency *ω*_2_ = 1 × 10^8^ MHz. The presented plots show clear modulation of amplitude of the wave with lower frequency *ω*_2_. The modulation of the amplitude of wave *ω*_1_ is not clear because amplitude of wave *ω*_2_ is weaker than wave *ω*_1_. To the contrary, we choose *ω*_2_ = 100 × 10^8^ MHz which is much higher than *ω*_1_ and the results are shown in figure 2(c). In this case, wave *ω*_1_ is clearly affected by wave *ω*_2_. There is modulation of the amplitude of wave *ω*_1_ which similar to the oscillation of a wave packet. The oscillation frequency is *ω*_2_. Wave *ω*_2_ is also affected by wave *ω*_1_. There is fluctuation of amplitude at a frequency *ω*_1_ and the amplitude of wave *ω*_2_ is repressed for most *r*. Figure 2(b) shows interactions between two waves with frequencies are close together. Modulations of the amplitudes are still existing, however, not clearly due to the small differences between the modulation frequency and the natural frequency of the waves.

One also can consider the case of more fundamental electromagnetic waves propagation in the nonlinear medium, and there are abundant nonlinear wave-wave interactions. Here, as an example, we consider the three fundamental waves case, and show that sum- and difference-frequency generation of two waves propagation in the nonlinear medium is affected by a third wave.

Equations (5) in the three waves case can be expressed as:


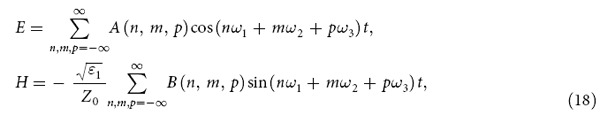


where *ω*_1_, *ω*_2_, and *ω*_3_ are frequencies of the three waves, and the electric field strength of the wave with frequency *ω*_*i*_ is recorded as *E*_*i*_. *A*(*n*,*m*,*p*) and *B*(*n*,*m*,*p*) can be obtained easily from Eqs. (6). Equations (18) contains a lot of frequency-mixing effects, while here we focus on the modification of sum- and difference-frequency generation in the presence of a third wave.

#### (i) Modification of sum-frequency generation

In this case, *p* = 0, *m* = *n* = 1 or *m* = *n* = −1 describes the sum-frequency generation process, so the amplitude of the sum-frequency generation can be obtained as *E*_*ω*1+*ω*2_ = *A*(1,1,0) + *A*(−1,−1,0).

**Figure 3:**
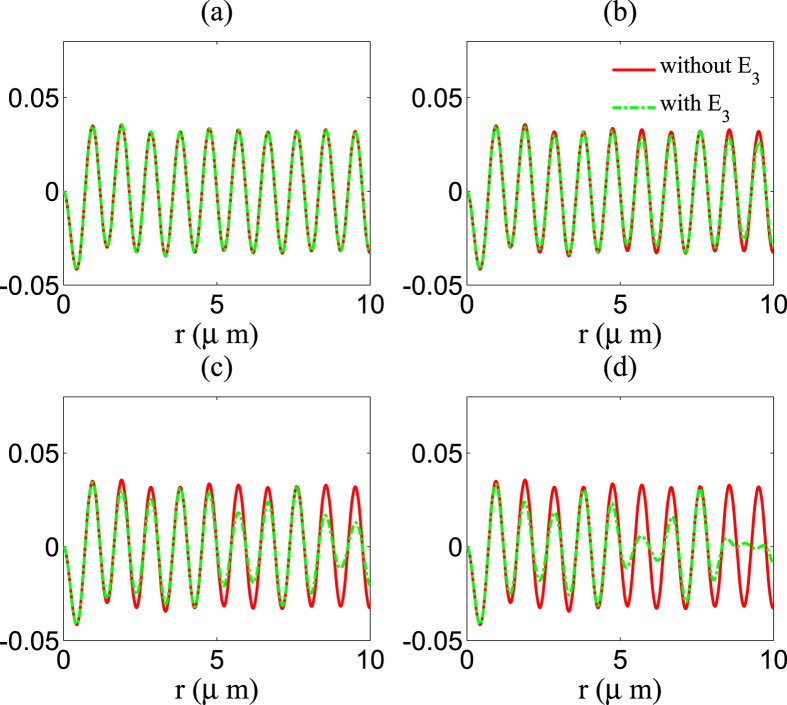
Sum-frequency generation of two waves propagation in the nonlinear medium is affected by a third wave, and the modification is increasing with the amplitude of the third wave. We consider that there are three waves propagation in the medium, and we use *ζ*_1_ = 0.5, *ζ*_2_ = 0.5, *α* = 0.4, and *ε*_1_ = 2 in equations (6). The red solid curve presents calculation result of sum-frequency generation *E*_*ω*1+*ω*2_ in the nonlinear medium without a third wave, the green dotted curve presents calculation results of *E*_*ω*1+*ω*2_ under a third wave with amplitudes (**a**) *ζ*_3_ = 0, (**b**) *ζ*_3_ = 0.5, (**c**) *ζ*_3_ = 1, (**d**) *ζ*_3_ = 1.5. We fix *ω*_1_ = 9 × 10^8^ MHz, *ω*_2_ = 5 × 10^8^ MHz, *ω*_3_ = 2 × 10^8^ MHz.

Figure 3 shows the calculation results of modification of sum-frequency generation in the presence of a third wave (the green dotted curve). We also plot the sum-frequency generation *E*_*ω*1+*ω*2_ in the nonlinear medium without a third wave by using a red solid curve. In Fig. 3(a), the amplitude of the third wave is zero, viz. *ζ*_3_ = 0, so there is no modification, and these two lines coincide with each other. In Fig. 3(b), the amplitude of the third wave is *ζ*_3_ = 0.5. There is a little modification, which, however, is very small. In Fig. 3(c) and Fig. 3(d), the amplitude of the third wave are *ζ*_3_ = 1 and *ζ*_3_ = 1.5, respectively. The modification is obvious, and there is a big gap between the two lines at some special *r*.

#### (ii) Modification of difference-frequency generation

In this case, *p* = 0, *m* = −*n* = 1 or *m* = −*n* = −1 describes the difference-frequency generation process, so the amplitude of the difference-frequency generation can be obtained as *E*_*ω*1−*ω*2_ = *A*(1,−1,0) + *A*(−1,1,0).

**Figure 4:**
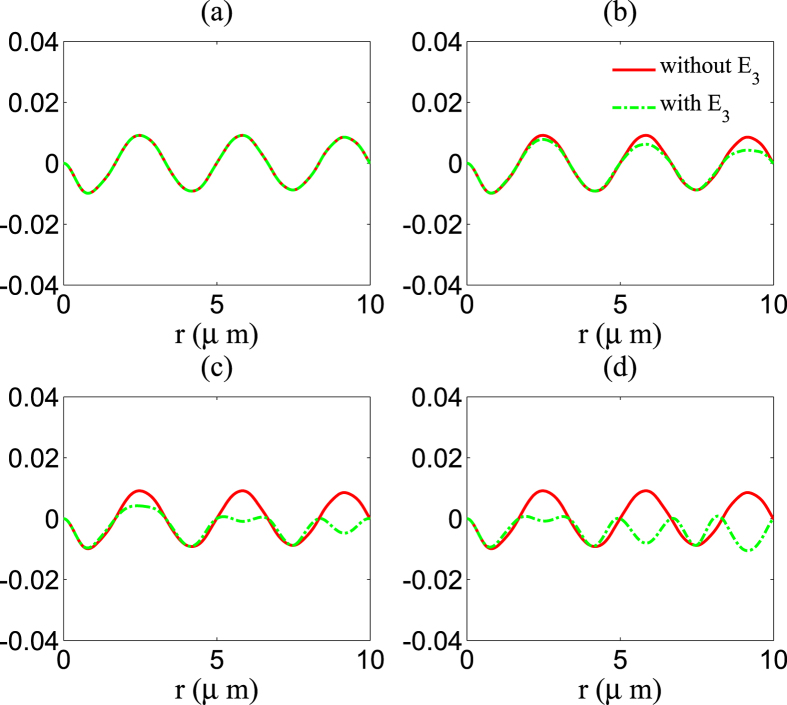
Difference-frequency generation of two waves propagation in the nonlinear medium is affected by a third wave, and the modification is increasing with the amplitude of the third wave. We consider that there are three waves propagation in the medium, and we use *ζ*_1_ = 0.5, *ζ*_2_ = 0.5, *α* = 0.4, and *ε*_1_ = 2 in equations (6). The red solid curve presents calculation result of difference-frequency generation *E*_*ω*1−*ω*2_ in the nonlinear medium without a third wave, the green dotted curve presents calculation results of *E*_*ω*1−*ω*2_ under a third wave with amplitudes (**a**) *ζ*_3_ = 0, (**b**) *ζ*_3_ = 0.5, (**c**) *ζ*_3_ = 1, (**d**) *ζ*_3_ = 1.5. We fix *ω*_1_ = 9 × 10^8^ MHz, *ω*_2_ = 5 × 10^8^ MHz, *ω*_3_ = 2 × 10^8^ MHz.

Figure 4 shows the calculation result of difference-frequency generation modification in the nonlinear medium in the presence of a third wave (the green dotted curve). Difference-frequency generation in the nonlinear medium without a third wave is also plotted by using a red solid curve. In Fig. 4(a), the amplitude of the third wave is zero, so there is no modification either. In Fig. 4(b), the amplitude of the third wave is *ζ*_3_ = 0.5. There is some modification, although not very large. In Fig. 4(c), the amplitude of the third wave are *ζ*_3_ = 1. The modification is obvious. For *r* ≈ 9 *μ*m, direction of the difference-frequency field *E*_*ω*1−*ω*2_ is even changed. It is much easier to see such phenomenon under a stronger *E*_3_. In Fig. 4(d), the amplitude of the third wave is *ζ*_3_ = 1.5. We can find that the direction of the difference-frequency field *E*_*ω*1−*ω*2_ is inverted near *r* ≈ 6 *μ*m and *r* ≈ 9 *μ*m.

The above discussions is based on Eqs. (18), and show that the sum- and difference-frequency generation of two waves propagation in the nonlinear medium can be greatly affected by a strong third wave. One also can discuss the modification of the sum- and difference-frequency generation in the presence of more additional waves by using the same way.

Our previous works^22^,^23^ preliminarily studied how to find and examine the physical nature contains in the new exact solution put forward by Petrov and Kudrin recently. A previous work^22^ studies the problem of single cylindrical wave propagation in a nonlinear medium, and finds that the second-harmonic generation comes out quite naturally from the exact solutions. Another previous work^23^ studies two cylindrical waves propagation in a nonlinear medium, and finds that the sum- and difference-frequency generation comes out quite naturally from the exact solutions. In the present work, we present a set of mathematical methods to deal with interactions between any amounts of waves in a nonlinear medium. For the case of three or more fundamental electromagnetic waves propagating in the nonlinear medium, there are abundant nonlinear wave-wave interactions. The modification of the sum- and difference-frequency generation in the presence of a third wave can be calculated easily by using the explicit analytical expression obtained.

Before ending this section, we would emphasize the notable merits of such reliable analytical method include:

1. We give an explicit analytical expression which contains all the main nonlinear optical effects, including second-harmonic generation, sum- and difference- frequency generation, electro-optical effect and waves mixing. The traditional method describing nonlinear optical effects is the coupled-wave-equations approach which can be solved only numerically in the cylindrical geometry. It is very difficult to give an explicit analytic expression which contains all the main nonlinear optical effects by using the coupled-wave equations.
2. Such an explicit analytical expression can deal with the problem of any amount of cylindrical electromagnetic waves propagation in a nonlinear medium. It is very difficult to deal with this problem by using the traditional coupled-wave-equations method^1^.
3. Our work is an interesting extension of the recent publication^19^, and deepening the understanding of the exact solution. From a solution of waves in a linear medium, one can obtain the solution of waves in a nonlinear medium through a simple variable substitution: *ρ* → *ρe*^*αE*/2^, 
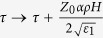
. Starting from the exact solution, we present a simple but highly efficient approach to deal with the interaction of any amount of cylindrical electromagnetic waves in a nonlinear medium. Description of interactions between a large amount of cylindrical electromagnetic waves in a nonlinear medium is a very complex question^1^, especially analytic description.

## Methods

Equations (4) can be approached by using the following method. At first, we give the linear solution as the zeroth approximation of Eqs. (4), as follows:


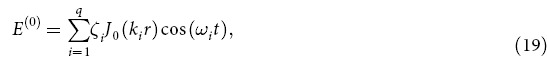



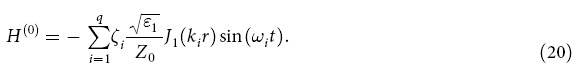


Substituting the zeroth approximation into Eqs. (4) leads the first approximation:


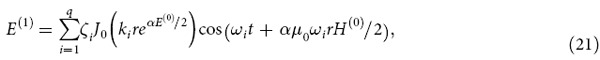



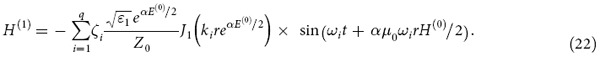


### Effective approximations

In this section we will give effective approximations of 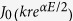 and 
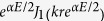
.


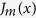
 is a Bessel function of the first kind of order 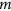 and can be presented as:


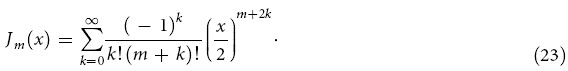


If 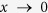, then Eq. (23) can be used to give an effective approximation of 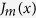 by series truncation. However, in the present case, 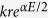 is not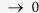 and in fact can be any number. So such series can not be used to give an effective approximation of our case^24^.

We take notice of *αE* is small, so we can write *J*_0_(*kre*^*αE*/2^) as:


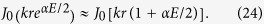


We introduce a function *f*_*n*_(*x*, *b*) as


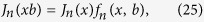


thus *f*_*n*_(*x*, 1) = 1 and


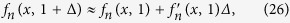
where 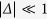 and 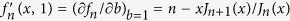. So
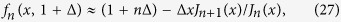


Using Eq. (25) and Eq. (27) we can give an approximation of 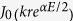 and 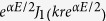. Following Eq. (24) we can write 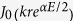 as:
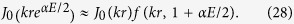


Using Eq. (27) we obtain:
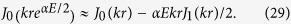
Similarly one can get:
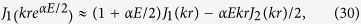
and
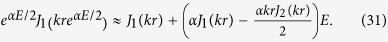


### Some identical equations

Here we give some identical equations which is used in our work:
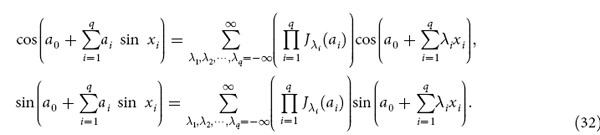


We demonstrate such identical equations from following identical equations:
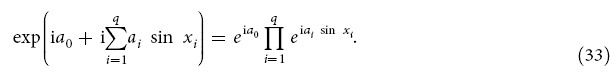


Using formula
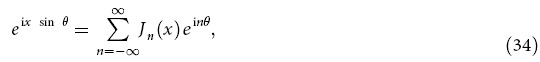


it can be find that:
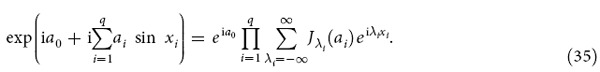


Exchange the sequence of summation and product, we can obtain:
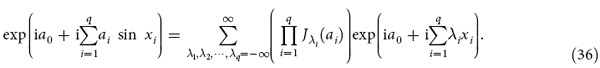


Take the real part and imaginary part of Eq. (36) leads Eqs. (32).

In what follows we will use Eqs. (32) to simplify some expressions, for example, 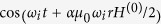 and 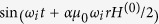. Substituting Eq. (20) into expressions leads:
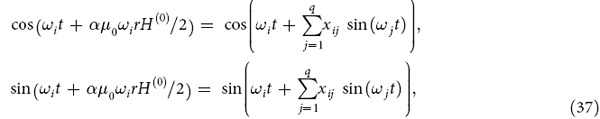
where *x*_*ij*_ have been defined as 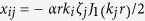. Using Eqs. (32) we can obtain:
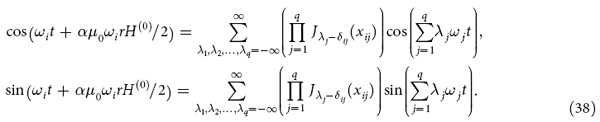


Using the same method we can simplify 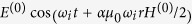 and 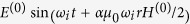. Here we give the results directly:
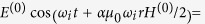

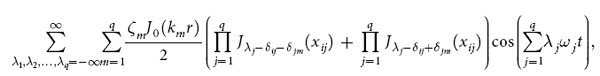

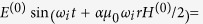

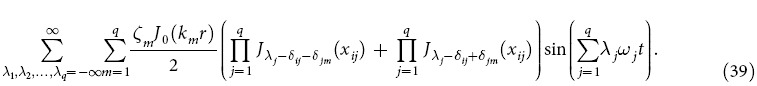


### Derivation of explicit analytical expressions

Equations (21) and (22) can be simplified by using approximations :
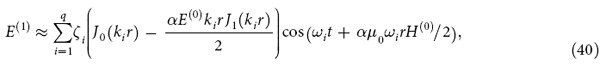

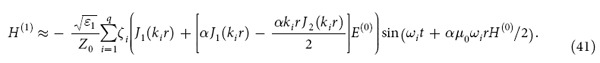


These equations can be easily rewritten as:
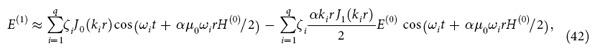

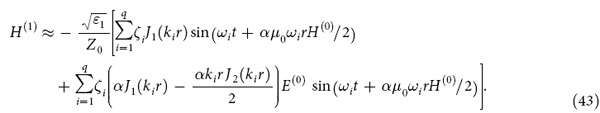


Substituting identical equations (38) and (39) into Eqs. (42) and (43), we can obtain:
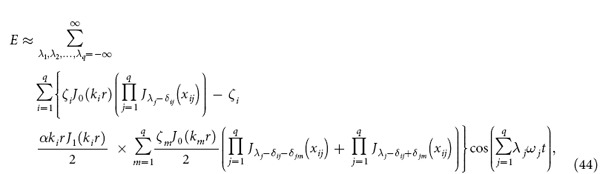

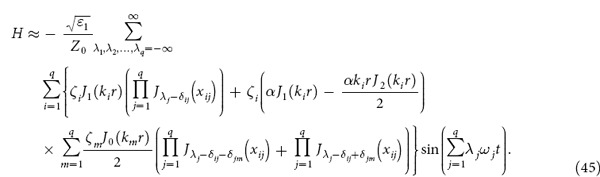


These expressions are exactly Eqs. (5).

## Additional Information

**How to cite this article**: Xiong, H. *et al.* Analytic descriptions of cylindrical electromagnetic waves in a nonlinear medium. *Sci. Rep.* **5**, 11071; doi: 10.1038/srep11071 (2015).
